# Dynamic changes of the gut microbial colonization in preterm infants with different time points after birth

**DOI:** 10.3389/fmicb.2023.1078426

**Published:** 2023-02-17

**Authors:** Adnan Khan, Hongying Mi, Fei Gao, Qi Hu, Xia Gu, Fei Ma, LiuHong Qu, Sitao Li, Yiheng Dai, Hu Hao

**Affiliations:** ^1^Guangdong Laboratory of Lingnan Modern Agriculture, Genome Analysis Laboratory of the Ministry of Agriculture and Rural Affairs, Agricultural Genomics Institute at Shenzhen, Chinese Academy of Agricultural Sciences, Shenzhen, China; ^2^Department of Pediatric, The First People’s Hospital of Yunnan Province, Kunming, Yunnan, China; ^3^Section of Comparative Pediatrics and Nutrition, Department of Veterinary and Animal Sciences, Faculty of Health and Medical Sciences, University of Copenhagen, Frederiksberg, Denmark; ^4^NEOMICS Institute, Shenzhen, China; ^5^Department of Pediatrics, The Sixth Affiliated Hospital, Sun Yat-sen University, Guangzhou, Guangdong, China; ^6^Department of Pediatrics, Zhuhai Women and Children's Hospital, Zhuhai, China; ^7^Department of Neonatology, The Maternal and Child Health Care Hospital of HuaDu District, Guangzhou, China; ^8^Department of Neonatology, Affiliated Foshan Maternity and Child Healthcare Hospital, Southern Medical University, Foshan, Guangdong, China

**Keywords:** preterm infants, gut microbiota, *Bifidobacterium*, *Escherichia coli*, *Klebsiella*

## Abstract

Risks associated with preterm birth are unevenly distributed across all gestations. At earlier gestational ages, complications such as necrotizing enterocolitis (NEC) and late-onset sepsis (LOS) conditions are significantly more common and are associated with a shift in the composition of the gut microbiome. Conventional bacterial culture techniques demonstrate that the colonization of the gut microbiota of preterm infants differs significantly from that of healthy-term infants. The current study aimed to investigate the impact of preterm infancy on the dynamic changes of fecal microbiota in preterm infants at different time points (1, 7, 14, 21, 28, and 42 days) after birth. We selected 12 preterm infants hospitalized in the Sixth Affiliated Hospital of Sun Yat-sen University from January 2017 to December 2017. A total of 130 fecal specimens from preterm infants were analyzed using 16S rRNA gene sequencing. We found that the colonization process of fecal microbiota in preterm infants is highly dynamic at different time points after birth, i.e., *Exiguobacterium*, *Acinetobacter,* and *Citrobacter* showed a declining abundance pattern with the advancement of age, while the bacterial groups of *Enterococcus* (*Klebsiella* and *Escherichia coli*) gradually grew and became the main microbiota during the development of fecal microbiota in preterm infants at the age of 42 days. Furthermore, the colonization of intestinal *Bifidobacteria* in preterm infants was relatively late and did not rapidly become the predominant microbiota. Moreover, the results also showed the presence of *Chryseobacterium* bacterial group, whose colonization was different in different time point groups. Conclusively, our findings deepen our comprehension and offer new perspectives on targeting particular bacteria in the treatment of preterm infants at different time points after birth.

## Introduction

Preterm birth is an important cause of neonatal morbidity and mortality in the perinatal period (Wiedmeier et al., 2011), and these effects are related to the gut microbiota, regulation of nutrient metabolism, developmental programming, and epigenetic modifications. About 1.5 million preterm babies are born every year in the country, especially after the opening of the two-child policy, a large number of advanced mothers become pregnant through *in vitro* fertilization combined with embryo transfer technology (IVF-ET), which dramatically increases the risk of preterm birth, and the number of preterm babies is increasing. The gut microbiota is involved in a variety of interactions that affect the host health throughout his life cycle (Cox et al., 2014), and colonization occurs in the previously sterile gut of a newborn. Interactions with colonizing intestinal bacteria are important for healthy intestinal and immune maturation in infancy. The establishment and growth of an infant’s gut microbiome have been shown to have vital effects on their short- and long-term health (Tauchi et al., 2019; Kim et al., 2020).

The diversity and richness of the fecal microbiota continue to differentiate with the development of nurslings until the age of 18 ~ 24 months (Collado et al., 2016), where its gut microbiota resemble the extremely dense and complex adult microbiota (Matamoros et al., 2013). Many theories have been proposed to explain where and how gut microbes are established, but most are still controversial (Lozupone et al., 2012; Ferretti et al., 2018). Although the composition of the gut microbiota in healthy infants has been extensively investigated, the dynamic changes in the gut microbiota of preterm infants in early ages have not been well-documented. The reasons are mainly related to the counting methods used, the low frequency of sample analysis, and the small number of subjects studied. In addition, intestinal microbiota engraftment in babies is more susceptible to the interference of some common factors (Yatsunenko et al., 2012). These factors include events such as gestational age (term versus preterm), delivery way (vaginal delivery versus cesarean section), premature rupture of membranes (mother or infant), use of antibiotics, feeding (breast milk versus formula), delayed enteral feeding, physiological maturity of infants, and body care after birth (mother’s care at home versus neonatal intensive care unit (NICU; Depner et al., 2020; Uzan-Yulzari et al., 2021). Thus, the investigation of factors related to intestinal bacterial colonization of preterm infants, timely detection of bacterial colonization, and appropriate measures will directly impact the quality of life of preterm infants.

Perturbations to the infant gut microbiome during the first weeks to months of life affect growth, development, and health (Favier et al., 2002). In particular, immunological and metabolic illnesses are more likely to occur if the infant’s gut microbiota is disrupted throughout development. This risk can last through childhood and even into adulthood (Furusawa et al., 2013; Healy et al., 2022). The gut microbiota of extremely preterm infants is dominated by one or more of the following: *Staphylococcus*, *Klebsiella*, *Enterococcus,* or *Escherichia*, and the predominance of one genus over another may periodically change (Stewart et al., 2016, 2017; Rao et al., 2021). Rao et al. (2021) revealed that the majority of preterm infants initially harbored staphylococcal-dominated communities but subsequently shifted to a different community-dominance state. The authors postulate that a shift in community composition may result through interbacterial and bacterial–fungal interactions, specifically exploitative interactions.

The majority of research into the development of the gut microbiome has focused on full-term babies, but health-related outcomes are also imperative for preterm infants. The systemic physiological immaturity of extremely premature babies (born earlier than 32 weeks gestation) results in various microbiome-organ interactions, the mechanisms of which have yet to be explored or, in some cases even considered. This study aimed to compare the gut microbiota of preterm infants at different time points, with a particular emphasis on the identification and potential clinical consequences of preterm infant gut microbiota composition.

Following the objective of the study, we investigated the composition of the developing fecal microbiota using 130 fecal samples from 12 hospitalized preterm infants and measured fecal microbiota and clinical indications at six different time points (1, 7, 14, 21, 28, and 42 days after birth). Finally, we profiled the dynamic changes in the fecal microbiota of preterm infants. We found that the colonization process of fecal microbiota in preterm infants was highly dynamic at different time points after birth, i.e., *Exiguobacterium*, *Acinetobacter*, and *Citrobacter* gave a declining richness pattern with the advancement of age, while bacterial species such as *Klebsiella pneumoniae* and *Escherichia coli* can gradually grow and become the main microbiota during the development of fecal microbiota in preterm infants.

## Materials and methods

### Study participants and fecal sample collection

A total of 12 preterm infants were recruited into the study population hospitalized in the Sixth Affiliated Hospital of Sun Yat-sen University from January 2017 to December 2017. Stool samples from preterm infants were collected using a disposable sterile special stool collection tube at a total of six time points 1, 7, 14, 21, 28, and 42 days after birth (C1, C2, C3, C4, C5, and C6, respectively). Two different parts of stool samples were collected at each time point. Among them, seven cases of preterm infants completed the collection of stool samples at six time points, two cases of preterm infants completed the collection of five time points, and the other three cases of preterm infants completed the collection of 3–4 time points. The gestational age and the weights were 30.01 ± 1.05 weeks and 1367.5 ± 137.2 g, respectively. In addition, the mode of delivery, gender composition, feeding method, duration of antibiotics, and probiotic use, for the 12 preterm infants are shown in Table 1; Supplementary Table S1.

**Table 1:** General clinical data of 12 preterm infants.

Preterm baby’s name	CYZ	CYP	GX	GLM	HGF	JLJ	LYX	LPY	LPY	LQMN	LQMZ	WYH
Gestational age (weeks)	31 + 2(days)	30 + 3(days)	30 + 1(days)	29	29 + 6(days)	28 + 5(days)	30 + 2 (days)	29	29	31 + 4(days)	31 + 4(days)	29 + 2(days)
Birth weight (g)	1,320	1,360	1,440	1,290	1,440	1,150	1,270	1,190	1,380	1,540	1,630	1,400
Mode of delivery	Natural	Natural	C-section	Natural	C-section	C-section	C-section	C-section	C-section	C-section	C-section	C-section
Gender	F	M	M	M	M	M	F	F	F	F	M	F
Feeding method (Breast milk, Formula)	Formula	Breast milk	Formula	Formula	Breast milk	Breast milk	Breast milk	Breast milk	Breast milk	Breast milk	Breast milk	Breast milk
Duration of antibiotics use (Days)	48	22	27	22	35	37	36	41	53	17	18	33
Duration of probiotic use (Days)	45	29	0	0	22	48	28	39	52	12	19	34

C-section, cesarean section; M, male; F, female.

To observe the dynamic changes of fecal microbiota at various time points, the collected fecal samples were immediately sent to the laboratory on the same floor. The stool was divided into 2–4 tubes based on the amount collected, placed in sterilized Eppendorf tubes, and then stored in the laboratory –80°C refrigerator for further Uniform DNA extraction of fecal microbiota.

### 16S rRNA gene sequencing and bioinformatics analysis

To assess the composition of microbial communities from clinical samples, total genome DNA was extracted from fecal samples using PowerSoil® DNA Isolation Kit (MOBIO, United States). The concentration of DNA was measured by Qubit® 3.0 Fluorometer (Invitrogen, United States), and purity was monitored on 1% agarose gels. The V3–V4 region of the bacterial 16S rRNA gene was amplified with barcoded primer 515F (GTGCCAGCMGCCGCGGTAA) and 907R (CCGTCAATTCMTTTRAGTTT). Samples with a bright main strip between 300 and 400 bp were chosen for further experiments. Sequencing libraries were generated using TruSeq® DNA PCR-Free Sample Preparation Kit (Illumina, United States) following the manufacturer’s instructions, and index codes were added. Finally, the library was sequenced on an Illumina NovaSeq platform (E-gene, Shenzhen, China), generating 250 bp paired-end reads in FASTQ format, and the corresponding paired-end reads were merged into a fragment. Version 2020.8 of the Quantitative Insights into Microbial Ecology 2 (QIIME2; Bolyen et al., 2019) pipeline^1^ was applied for sequence quality assessment and de-noise using the Divisive Amplicon Denoising Algorithm 2 (DADA2; Callahan et al., 2016). After filtering, short sequences (<100 nt) and bases at the end of the sequence with lower quality (<20) were excluded. Both forward- and reverse-sequencing reads after trimming met the criteria were retained for analysis.

### Taxonomy assignment

The filtered reads were taxonomically classified into OTU against the 99% identity SILVA (release 119) V3–V4 classifier (Quast et al., 2013). All the ribosomal sequence variants (RSVs) were identified as features across all samples without clustering. The feature table rooted phylogenetic tree, representative sequences, and metadata from QIIME2 were then exported for further analysis in R version 3.4.2^2^. For Alpha diversity analysis demonstrating the complexity of species diversity, QIIME2 was used to compute the evenness index and the observed operational taxonomic units (OTUs) index, and the results were visualized with R. Moreover, the different tests of Alpha diversity for different groups were performed using Wilcoxon Rank Sum Test. Beta diversity was calculated using Bray–Curtis distance and Weighted UniFrac distance by the R package VEGAN version 2.5–3^3^ (Oksanen et al., 2015). Differences in beta diversity were identified using Analysis of Similarity (ANOSIM), and the effect size was indicated by an R-value (between −1 and +l, with a value of 0 representing the null hypothesis) (Clarke, 1993), and PERMANOVA test leveraged by stress and effect size *R*^2^ between 0 and 1. Principal coordinate analysis (PCoA) from the R package ape^4^ and the non-metric multidimensional scaling (NMDS) method from the R package VEGAN were used to visualize differences in the beta diversity-based community structure. Linear discriminant analysis (LDA) effect size (LefSe) analysis was performed to reveal the significant ranking of abundant modules in six time point groups (Segata et al., 2011). A size-effect threshold of 2.5 on the logarithmic LDA score was used for discriminative functional biomarkers. Significantly different biomarkers at phylum and genus levels were identified using STAMP (v2.1.3)^5^ (Parks et al., 2014).

### Prediction and identification of metagenome functional content and biomarkers

To further study the biological function of metagenomics, The Phylogenetic Investigation of Communities by Reconstruction of Unobserved States (PICRUSt v1.1.4)^6^ software was employed to predict the functional composition of metagenomes bases on OTU table (Ashauer et al., 2015). Gene abundances in metabolic pathways were analyzed using the KEGG groups. OTU data generated in QIIME for all 16S rRNA datasets were used to build BIOM files formatted as input for PICRUSt, and PICRUSt-predicted metagenomes based on OTUs marker gene sequences were estimated using default parameters. We furthered our study by detecting the principal component analysis (PCA) of the PICRUSt-predicted KEGG abundance of all samples using R package ade4^7^. The significantly different (*p* < 0.05) biomarkers, including KEGG pathway, were identified by LEfSe (Segata et al., 2011), using selection criteria of alpha value for the factorial Kruskal–Wallis test of 0.05 and the linear discriminant analysis (LDA) score of >2.5. The statistical significance for all analyses was set as *p* < 0.05.

### The interactive networks of gut microbiota

**Figure 1:**
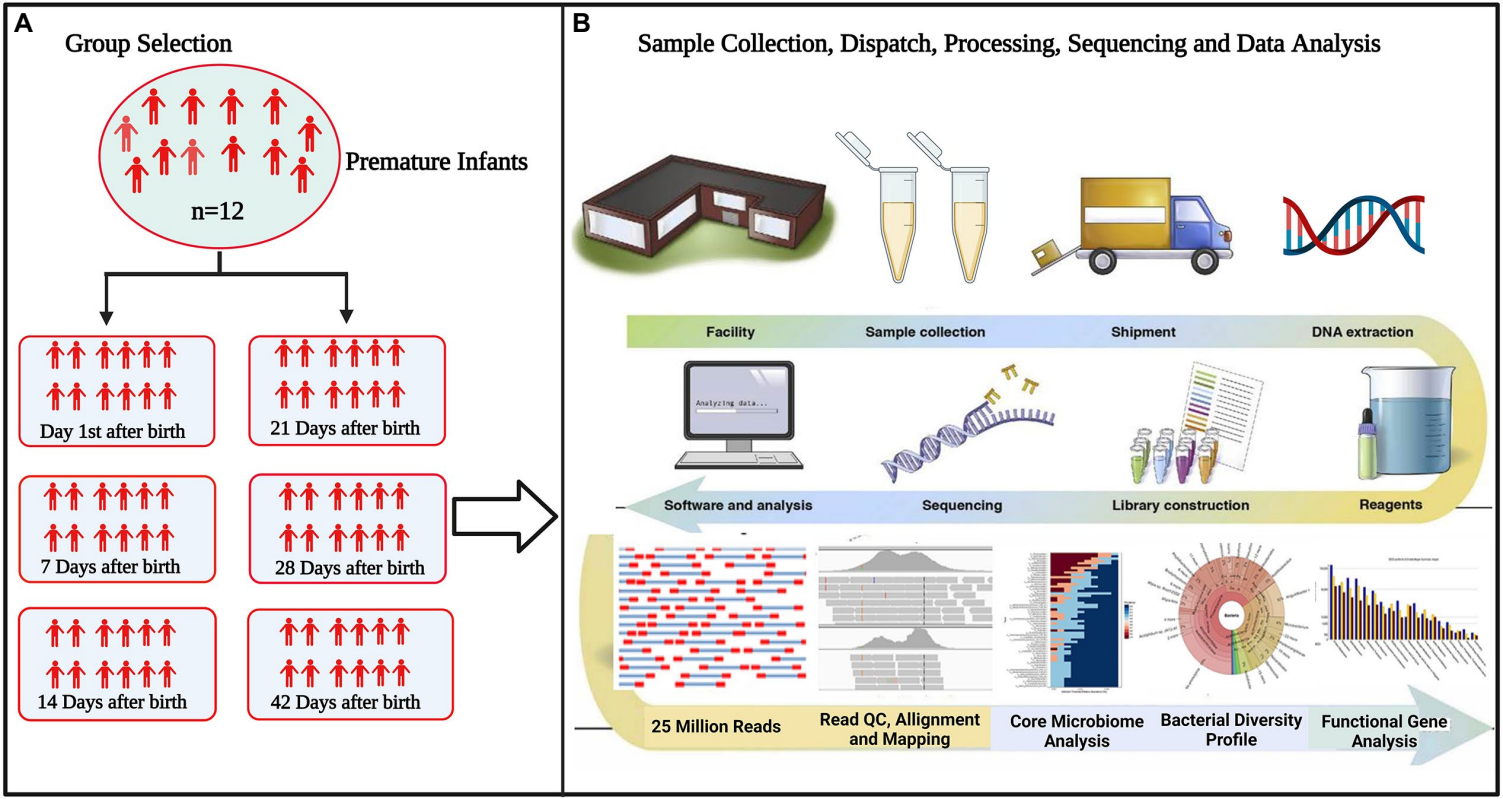
Flowchart of the study **(A)** selection of experimental groups to determine differences in microbiota between groups **(B)** standardizing technical factors and sample processing to control for variation introduced by every step of the process. Data interpretation necessitates the collection and curation of metadata about all aspects of each sample, from clinical variables to sample processing.

The correlation network has an absolute Pearson’s correlation above 0.50 with a significance level under 0.05, and these correlations were transformed into links among genera in the co-occurrence network using a self-develop Perl script (Che et al., 2019). The co-occurrence networks were then visualized using Cytoscape version 3.9.1.^8^ The schematic overview of the overall methodology is shown in Figure 1.

### Statistical analysis

The Shannon’s diversity index was calculated to measure species richness and distribution evenness in the community, and the Chao1 index estimated the total number of OTUs based on the actual observed species number. The Mann–Whitney non-parametric test was used to measure the differences between continuous variables of two groups and Kruskal–Wallis for more than two groups with a significance threshold of 0.05. Additionally, One-way analysis of similarity (ANOSIM) was used to evaluate for statistically significant differences between metagenomic profiles. *p*-values ≤0.05 were considered significant.

## Results

### Comprehensive characterization of microbial community composition in preterm infants

We used 16S rRNA gene data of 130 fecal samples from 12 preterm infants at multiple time points (C1–C6) after birth to assess time-related changes in microbial diversity, identify the top abundant and enriched drivers of microorganisms in different time point groups, and explore the phenotypes and function of fecal microorganisms during the healthy development of preterm infants.

**Figure 2:**
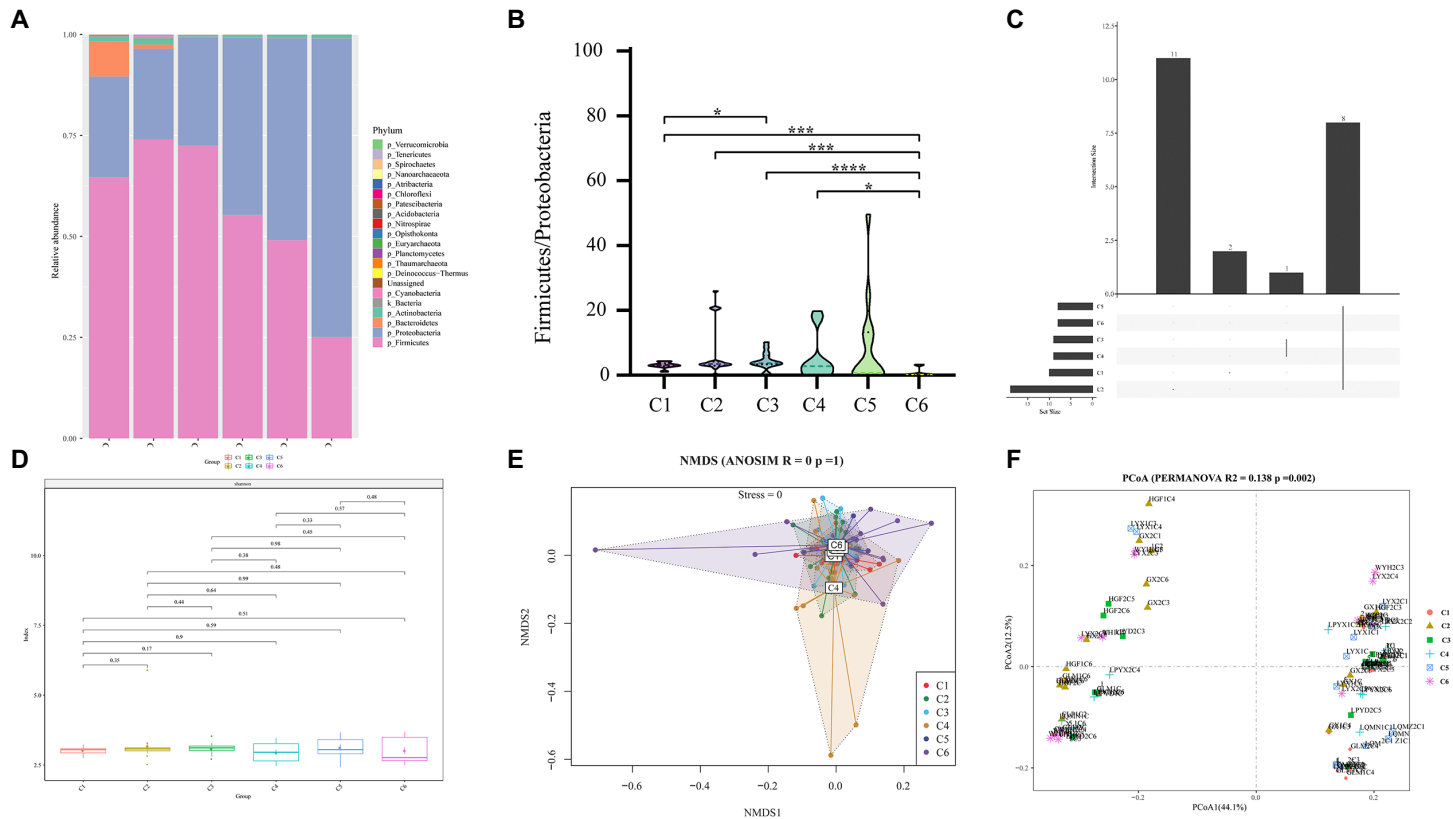
Comparison of relative abundances of intestinal microbial taxa across 6 age time points of preterm infants. **(A)** Composition of gut microbiota at the phylum level in the 6 age time points. **(B)** Box plot of F/P ratio among all groups (C1–C6). **(C)** Venn plot illustrating overlap of gut microbial phyla among six time point groups. Phyla detected in more than six fecal samples are included. **(D)** Alpha diversity and richness of bacterial communities across six groups using the Shannon index. **(E)** Beta diversity using NMDS of Bray–Curtis dissimilarity. **(F)** Unweighted Unifrac distance of gut microbiota between the time point groups. Pairwise *p*-values are calculated using a non-parametric Kruskal–Wallis test with Tukey *post hoc* test. *p < 0.05, **p < 0.01, ***p < 0.001, ****p < 0.0001 were considered significant.

Sequencing metadata of fecal DNA 16S rRNA gene are summarized in Supplementary Table S2. Rarefaction analysis of the observed operational taxa (OTUs) showed that sequencing effectively captured the potential total OTUs in the stool samples (Supplementary Figure S1). The top five phyla observed in fecal specimens were *Firmicutes* (25.11–73.96%), *Proteobacteria* (22.51–73.86%), *Bacteroidetes* (0.02–8.74%), *Actinobacteria* (0.04–1.47%), and *Cyanobacteria* (0.01–0.34%) (Figure 2A), among which *Firmicutes* and *Proteobacteria* were the dominant phyla showing that the *Firmicutes* were predominant in C1–C3 groups, while the *Proteobacteria* were predominant in C4–C6 groups. Additionally, the ratio of *Firmicutes* to *Proteobacteria* (F/P) in feces was measured across all time points (C1–C6), revealing a significant (*p* < 0.05) difference in the F/P ratio between groups. It is worth highlighting that the C5 (28 days) group had significantly (*p* < 0.05) a higher F/P ratio than all other groups (Figure 2B). The Venn plot in Figure 2C shows that 8 (19.69%) were shared across different time points.

Furthermore, a comparison of Shannon diversity indexes for all time points (C1–C6) is shown in Figure 2D (also see Supplementary Table S3). The indices were shown in all groups and found a subsequent increase with the advancement of age, but the differences were not significant. Likewise, Pielou’s evenness, observed OTUs, faith_pd (Supplementary Figure S2), chao1, dominance, and Simpson index (Supplementary Table S3) showed no significant change in alpha diversity among the six time point groups (C1–C6). For beta diversity, in the NMDS based on the Bray–Curtis distance matrix, no obvious clustering was found in six different time point groups (Figure 2E). However, with time, the sample heterogeneity of microbiota structure in each group increased, and the parallelism within the group was poor. In addition, permutational multivariate analysis of variance (PERMANOVA) results based on unweighted UniFrac distance (*R*^2^ = 0.138, *p* = 0.002) indicated a significant difference among the six time point groups (Figure 2F). These results of Figure 2 thus pointed to remarkable microbial community changes associated with a timeline.

### The top abundant genera in gut microbiota of preterm infants at different time points after birth

**Figure 3:**
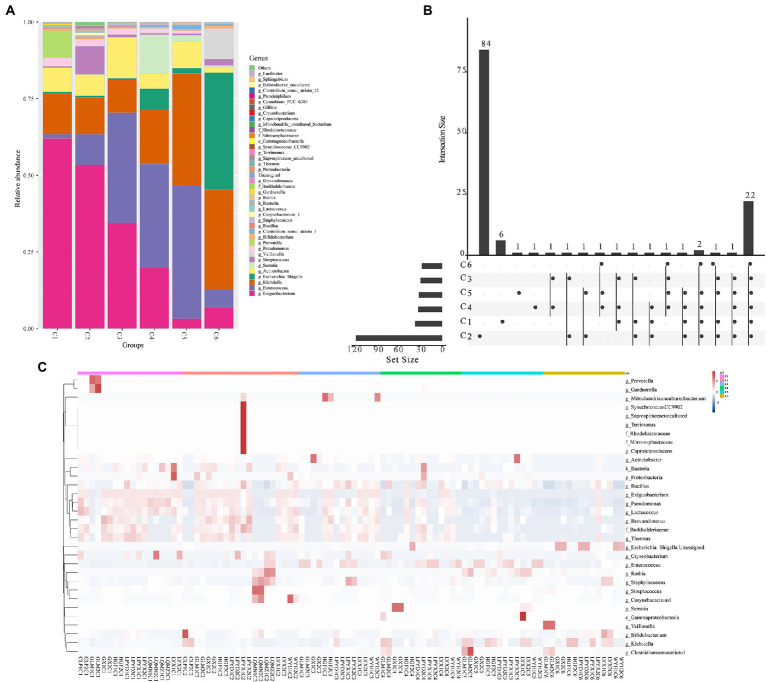
The top abundant genera of intestinal microorganisms enriched in 6 age time point groups of preterm infants. **(A)** Composition of the top 40 most abundant genera in the six time points (C1–C6). **(B)** Venn plot illustrating overlap of gut microbial genera among 6 age groups. **(C)** Heatmap showing the most abundant differentially expressed genera in the gut microbiota of the six time point groups. Single letters (g, f, and c) in front of names indicate the genus, phylum, and class, respectively.

The findings of the investigation into the most abundant genera were consistent with the beta diversity, suggesting that the diversity of the most abundant genera increased over time. These results revealed a time-dependent shift in the most abundant genera (*Exiguobacterium, Prevotella, Acinetobacter, Pseudomonas, Enterococcus, Bifidobacterium, Escherichia–Shigella, Klebsiella, Gardnerella, Streptococcus,* and *Chryseobacterium*) (Figure 3A), as well as the sample heterogeneity of the floristic structure of each group increased. Figure 3A shows the top 40 most abundant genera in each time group, half of which were common to all time groups (Figure 3B). In addition, the heatmap in Figure 3C indicated by cluster analysis that fecal microbiota in C1–C3 was clustered of *Exiguobacterium, Pseudomonas, Lactococcus, Brevundimonas, Burkholderiaceae,* and *Thermus*; in C3–C5 was clustered of *Enterococcus*, and in C6 was clustered of *Escherichia − Shigella*.

### Differential taxa of gut microbiota across 6 age groups of preterm infants

**Figure 4:**
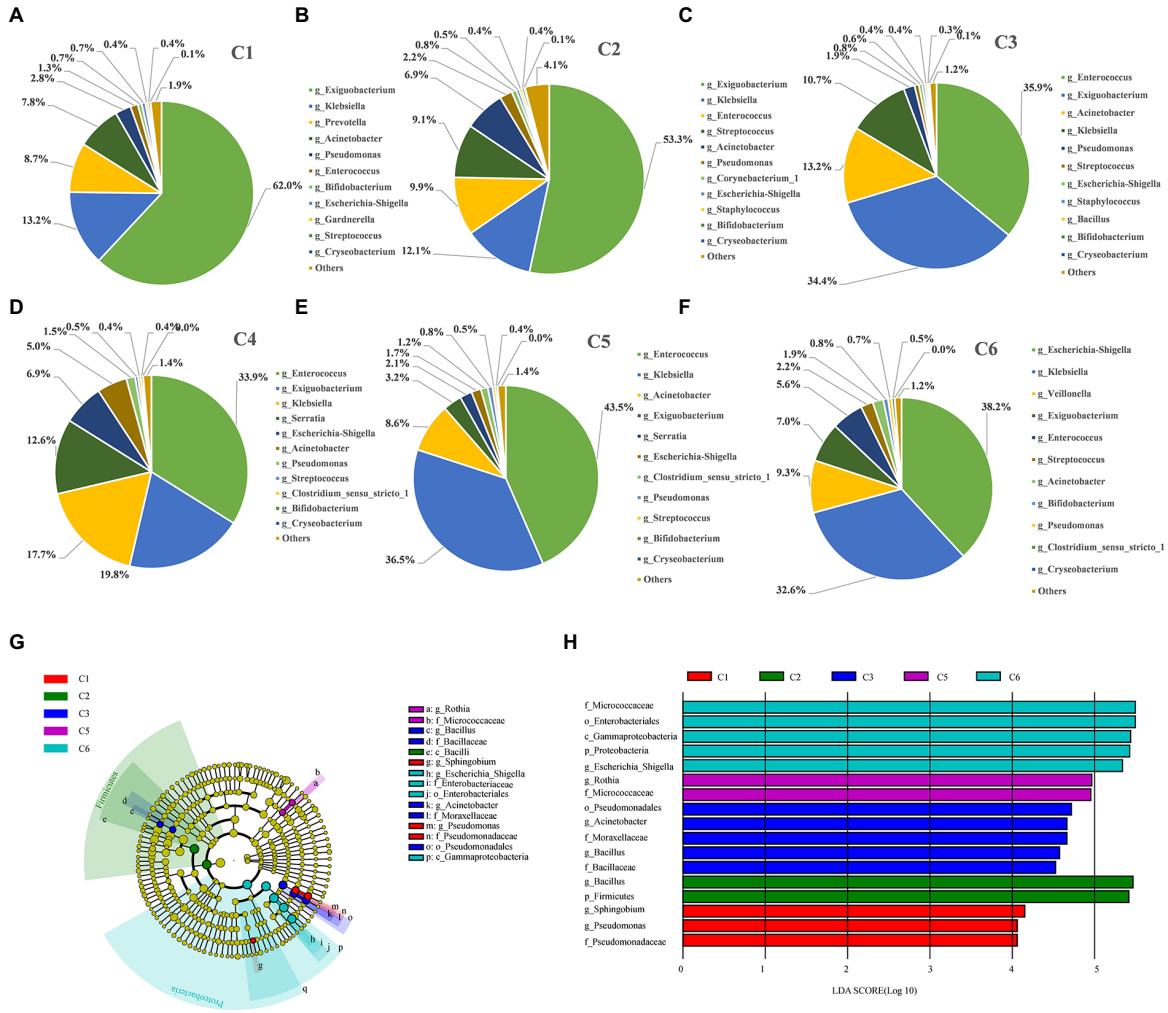
Figure Differentially abundant taxa enriched in the six time point groups of preterm infants. **(A–F)** Dynamic changes of dominant microbes at genus level in the intestinal tract of preterm infants at different time points. **(G)** Phylogenetic cladogram showing differentially abundant taxa from phylum to genus levels. Microbial classes are indicated with letters. Each node represents one taxon at different taxonomic levels. Node colors are the taxa that were observed with higher abundance in the relevant cohort (C1–C6). **(H)** Species with a significant difference have an LDA score greater than the estimated value; the default score is 2.5. The length of the histogram represents the LDA score; i.e., the degree of influence of species with significant differences between different groups.

To further characterize the dynamic gut microbiota changes during six time point groups. We did time point-dependent distribution of phylogenetic shifts of these taxa at different taxonomic levels, i.e.; genus (Figures 4A–F), phylum, class, order, family, and species (Supplementary Figure S3).

Furthermore, the most dynamically enriched microbiota among all groups were investigated at the genus level. The abundance of *Exiguobacterium* (62%), *Prevotella* (8.7%), and *Acinetobacter* (7.8%) was higher in the C1 group, and then gradually lowered with the advancement of age (C2–C6). However, the abundance of *Klebsiella* (13.2%) and *Escherichia–Shigella* (0.7%) was lower on day 1 (C1) but increased gradually over time to become the dominant taxa in C6 group by 32.6 and 38.2%, respectively (Figures 4A–F). Moreover, the abundance of the most dynamically enriched microbiota at the species level includes *Escherichia coli* and *Klebsiella pneumonia* giving ascending pattern from C1 to C6 group. Besides, *Bifidobacterium animalis* and drug-resistant bacterial strain *Chryseobacterium indologenes* showed a declining pattern from C1 to C6 (Supplementary Figure S3E; Supplementary Table S6). Moreover, through LEfSe, we determined the most abundant differential taxa in the six time point groups. At the phylum level, *Firmicutes* and *Proteobacteria* were the biomarkers for C2 and C6 groups, respectively, which was in compliance with the prior observation that *Proteobacteria* and *Firmicutes* predominated at the phylum level, with the decrease of *Firmicutes* and the increase of *Proteobacteria* over time (Figures 4G,H; Supplementary Table S4). No phylum was enriched in C4 group. The most abundant families and enriched genera were consistently found in C6 group. Additionally, *Pseudomonas* and *Sphingobium* were the most abundant genera in the C1 group. Two important groups of *Bacillus* and *Acinetobacter* were shown to be enriched in the C3 stage, among which *Bacillus* was pathogenic. Besides, *Rothia* was the most enriched in C5 group. A total of six taxa were enriched in C6 stage, including *Micrococcaceae*, *Enterobacterales*, *Gammaproteobacteria, Proteobacteria, Escherichia–Shigella, and Escherichia coli*.

### Age-dependent gut microbiota networks and their key driver genera in preterm infants

**Figure 5:**
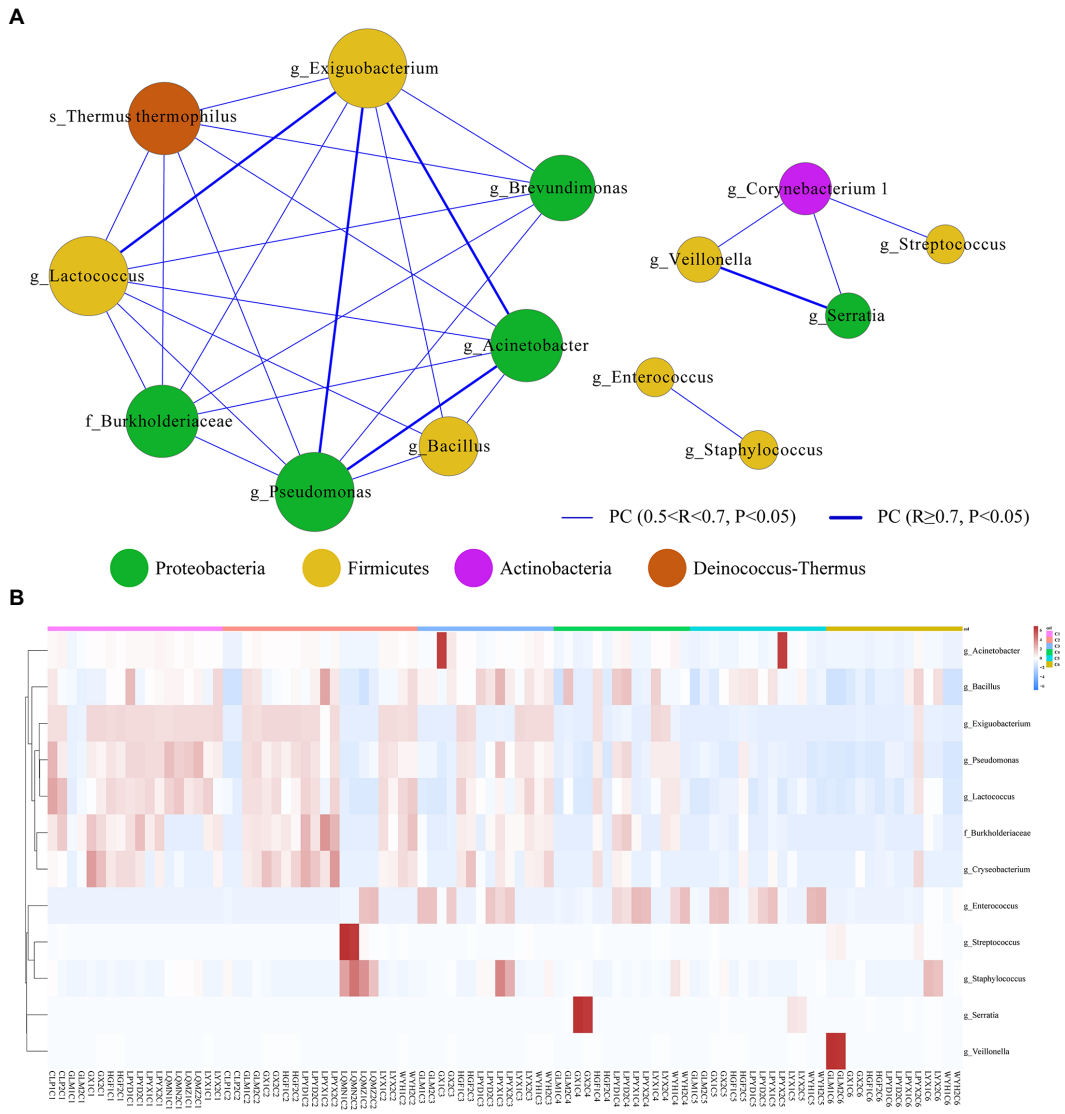
**(A)** Microbial interactive networks in preterm infants are constructed from SparCC results, and visualized using Cytoscape. (C1) Day 1st, (C2) 7 days, (C3) 14 days, (C4) 21, (C5) 28 days, and (C6) 42 days after birth. Genera with average abundance >0.1%, correlation |*R*| > 0.2, and *p* < 0.05 are included in the networks. Node colors denote the phyla of the genera. Node sizes represent weighted node connectivity. Edge colors and thickness represent the correlation. **(B)** Heatmap of top 12 differentially abundant microbial genera in all time-dependent samples (C1–C6). Red corresponds to an up-regulated gene product, and green corresponds to a down-regulated gene product. Each differentially expressed phylum is represented by a single row, and each sample is represented by a single column.

We then used the sparse compositional correlation (SparCC) analysis to explore the interaction among gut microbes in all time-dependent samples. All genera with relative abundance ≥0.1% were included in the networks. The *Exiguobacterium* (*Firmicutes*) network had the strongest connectivity with *Acinetobacter (Proteobacteria), Pseudomonas (Proteobacteria), and Lactococcus (Firmicutes).* Likewise, *Serratia (Proteobacteria)* is also strongly connected with *Veillonella* (*Firmicutes).* However, *Streptococcus, Staphylococcus, Enterococcus, and Bacillus (Firmicutes)* showed the weakest connectivity with the rest of the genera (Figure 5A). A heatmap and hierarchical clustering of the top 12 differentially abundant genera with |FC| > 1 and *p* < 0.05 demonstrates the relatedness of samples (Figure 5B).

### Prediction of microbial metabolic functions based on taxonomic composition

**Figure 6:**
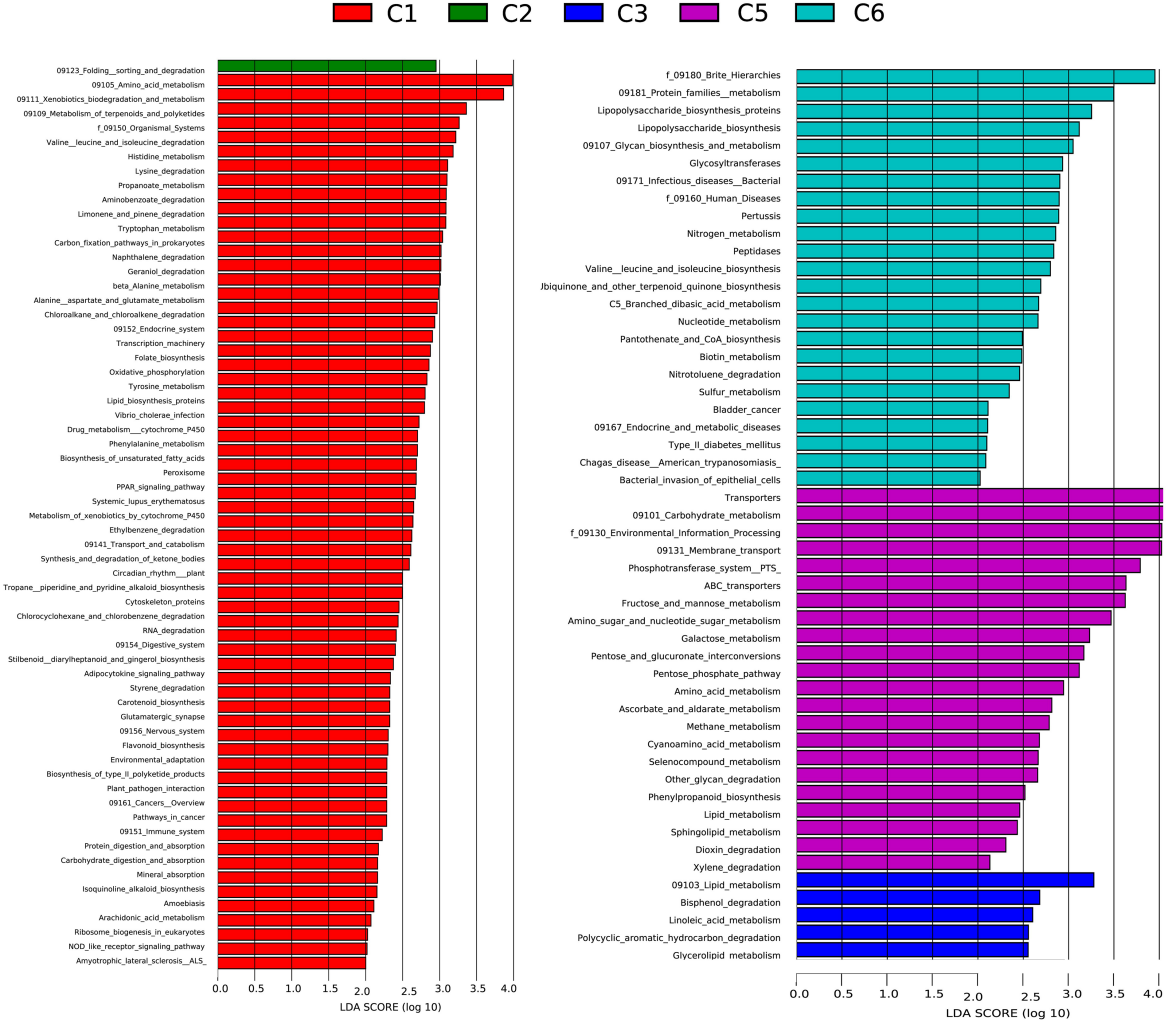
Comparison of relative abundance of functional modules among preterm infants at 6 time points C1, C2, C3, C5, and C6 (1, 7, 14,21, 28, and 42 days after birth) groups using Linear discriminant analysis effect size (LEfSe) analysis generated from PICRUSt. A number of 35, 1, 5, 18, and 15 KEGG modules are significantly enriched in C, C2, C3, C4, and C6, respectively. Data show differences in predicted bacterial metabolic function among all groups. Pathways with significant difference have an LDA score > 2.5.

To better understand the functional differences in the gut microbiome, disease susceptibility, and metabolic contribution of bacteria in preterm infants, a functional profile was generated using PICRUSt to predict gene families based on bacterial metagenomes by predicting genes from 16S rRNA data derived from generated OTUs and its reference genome database (Ashauer et al., 2015). The results showed significant overexpression of 63, 1, 5, 22, and 24 KEGG pathways in C1, C2, C3, C5, and C6 groups of preterm infants, respectively (Figure 6; Supplementary Table S5). Most of the pathways identified in the C1, C2, C3, and C5 groups are necessary for the sustenance of life, including ABC transporters, Nucleotide metabolism, Ascorbate, and aldarate metabolism, carbohydrate, and protein metabolism Dioxin degradation, Lipid metabolism. Finally, numerous modules describing metabolic processes and immune status were identified to be over-represented in all groups. All these modules are essential in affecting microbial distribution, survival, and proliferation of microbes in the environment (Supplementary Figure S4). These results would further need to be confirmed using metagenomics.

## Discussion

Preterm birth is a significant cause of neonatal morbidity and mortality. In preterm infants, colonization of the gut microbiota is important for the pathogenesis of, for example, LOS and NEC, two major causes of mortality. Microbial transfer from the gastrointestinal tract has been suggested to cause LOS and NEC in very low birth weight infants (Sherman, 2010). LOS is a major cause of neonatal morbidity and mortality, especially in preterm infants. During the establishment and development of healthy gut microbiota, commensal microorganisms parasitizing the digestive tract may cause a mucosal barrier infection and spread throughout the body (Mai et al., 2013). The study by Luo Jun et al. from the Maternal and Child Health Hospital of Bao’an District, Shenzhen, found that the main pathogenic bacteria in blood culture of children with NEC above stage IIa combined with positive blood culture sepsis were *Klebsiella pneumoniae* (32%), *Escherichia coli* (32%), *Enterococcus faecalis* (12%), and *Enterococcus faecalis* (8%) (Backhed et al., 2015). Bizzarro et al. (2014) conducted an etiological analysis of 158 children with NEC complicated with sepsis and found that a very high proportion (51.6%) of children with sepsis within 72 h of NEC diagnosis were infected with *Klebsiella*. Wu et al. (2012) and other studies found that the Gram-negative bacteria in 56 preterm infants were mainly *Klebsiella* 25.58% and *Escherichia coli* (6.56%). Through the analysis of the fecal microbiota of 12 preterm infants, we found that *Klebsiella* and *Escherichia coli* significantly increased at about 7 days after birth and became higher and higher as time went on and became the main microbiota. *Klebsiella* bacteria and *Escherichia coli* were the predominant gut microbiota around 42 days after birth, with respective proportions of 32.6 and 38.2% (Figures 4A–F). It shows that some opportunistic pathogenic bacteria, such as *Klebsiella* and *Escherichia coli,* can gradually grow during fecal microbiota development in preterm infants. Similarly, opportunistic pathogens can easily invade the bloodstream through the intestinal mucosa to infect the body of preterm infants due to their low immunity and poor intestinal mucosal barrier function, causing severe damage to the body.

Conventional bacterial culture methods confirm that the gut microbiota colonization of preterm infants is significantly different from that of healthy-term neonates. Compared with healthy-term infants, probiotics such as *Bifidobacteria* that regulate intestinal development in preterm infants, including epithelial barrier integrity, tend to colonize the intestinal tract of preterm infants late and less frequently, takes almost 1 week before colonization begins (Tanaka et al., 2019). In the investigation of preterm infant’s fecal microbiota, we found that the colonization of intestinal *Bifidobacteria* in preterm infants was relatively late and did not rapidly develop into the dominant microbiota. The low abundance of *Bifidobacteria* in the gut microbiota of preterm infants thus increases intestinal epithelial permeability, making them prone to enteric diseases. Many interfering factors, such as differences in sex, way of delivery, and feeding, are confounding factors in the study of the factors affecting fecal microbiota colonization in preterm infants. However, the results show that the differentiation process of the fecal microbiota of premature infants is still highly consistent, and the distribution of the fecal microbiota of premature infants is mainly composed of *Exiguobacterium*, *Acinetobacter,* and *Citrobacter* around 1 day after birth, making their proportion as high as 83% of the total population. Over time, the content of these three main bacterial groups gradually decreased, while the bacterial groups of *Enterococcus*, *Klebsiella*, and *Escherichia coli* gradually increased and became the main bacterial groups by 42 days after birth.

In the past, gut microbiota studies have relied on bacterial culture methods. The bacterial culture method found that the main bacteria colonizing infants were *Streptococcus*, *Staphylococcus*, *Enterobacter*, and *Enterococcus* (Inoue and Shimojo, 2015). Backhed et al. (2015) performed a more comprehensive analysis of the gut microbiota of 98 infants and their mothers using metagenomic shotgun sequencing and found that *Firmicutes* and *Bacteroidetes* were the most common phyla, followed by *Actinobacteria* and *Proteobacteria*. Besides, several studies have noted that *Firmicutes* dominate the earliest stool samples of preterm babies (La Rosa et al., 2014; Rao et al., 2021). However, the bacterial load is probably very low at this stage of colonization. As feces production occurs, there is a marked shift to persistent *Proteobacteria* dominance (Young et al., 2020). *Enterobacteriaceae*, including *Klebsiella* and *Escherichia*, have a substantially higher relative abundance than *Bifidobacterium*, which exist at a much lower relative abundance than in term babies (Patel et al., 2016). We also found that in the early age groups (C1, C2, and C3), the main microbiota in preterm infants was *Firmicutes* (*Exiguobacterium*, *Bacillus*, *Veillonella*, *Streptococcus,* etc.) which then dominated by *Proteobacteria* (*Escherichia–Shigella, Pseudomonas, Acinetobacter, Citrobacter*, *Klebsiella,* etc.) in the subsequent age groups (C4, C5, and C6), and is not entirely consistent with the distribution of fecal microbiota in normal term infants. Moreover, Fallani et al. (2010) evaluated the intestinal microbiota of 42-day-old European infants. They found that the diversity of bifidobacteria in infant feces in northern European countries is comparatively higher. In contrast, the content of Bacteroides in infant feces in southern European countries is relatively high (Fallani et al., 2010), depicting that even in European populations, the fecal microbiota of infants is vastly different, and infants in each place have their dominant microbiota.

*Chryseobacterium* is a gram-negative bacillus that produces yellow pigment, non-human normal microbiota, and has multi-drug resistance properties. In the past, a mass outbreak occurred in the internal medicine intensive care unit (ICU) ward of a medical center, and seven people were infected with aureus (Kanamori et al., 2016). The investigation revealed that the personnel failed to take necessary precautions for hand hygiene and infection control (Cantero et al., 2018). In addition, there is also a case report of neonatal meningitis related to this bacteria (Maraki et al., 2009), suggesting that there may be drug-resistant bacteria in the NICU, which may become an important source of infection in NICU outbreaks. In the present study, we discovered that the fecal microbiota of preterm infants in NICU contain *Chryseobacterium indologenes*, which poses a threat to their lives. Current research can help us to learn more about the regulations of fecal microbiota in preterm infants by analyzing the dynamic changing process of colonization at different time points after birth.

## Conclusion

Our findings will contribute to gaining a better understanding of the long-term health consequences of postnatal changes in the gut microbiota of preterm infants. To our knowledge, this is the first study describing the composition of the fecal microbiome in preterm infants under 42 days of age. Fecal microbiota in preterm infants is more simplistic around a day after birth, with *Exiguobacterium*, *Acinetobacter*, and *Citrobacter* making up 83% of the entire microbiota. Over time, the relative abundance of these three major bacterial families declined, while the bacterial groups of *Enterococcus*, *Klebsiella*, and *Escherichia* gradually increased and became the main bacterial groups, any of which could cause a digestive tract infection. Similarly, the colonization of intestinal *Bifidobacteria* in preterm infants was relatively late and did not rapidly become the predominant microbiota. In addition, *Chryseobacterium* bacterial group exists in the fecal microbiota of preterm infants in NICU, indicating an additional risk to their health. We hope that the current study could open new possibilities for comparison and understanding of the time-dependent dynamics of the gut microbiota in preterm infants and offer new perspectives on targeting particular bacteria for the treatment of premature infants at different time points after birth.

## Data availability statement

The data presented in the study are deposited in the NCBI repository with accession BioProject ID: PRJCA014298.

## Ethics statement

The studies involving human participants were reviewed and approved by the Ethics Committee of the Sixth Affiliated Hospital of Sun Yat-sen University (Batch No. 2019ZSLYEC-080). Written informed consent to participate in this study was provided by the participants’ legal guardian/next of kin.

## Author contributions

AK and HM designed and conducted the microbiota investigation and wrote the original manuscript. FG, SL, YD, and HH designed the study and revised the manuscript. XG, FM, and LQ conducted the bioinformatics and statistical data analysis. All authors contributed to the article and approved the submitted version.

## Funding

This work was financially supported by the Science and Technology Planning Project of Guangdong Province, China (201704020086 and 202002030008), the Social Science and Technology Development Foundation of Dongguan, China (201950715023152), and the Agricultural Science and Technology Innovation Program of CAAS.

## Conflict of interest

The authors declare that the research was conducted in the absence of any commercial or financial relationships that could be construed as a potential conflict of interest.

## Publisher’s note

All claims expressed in this article are solely those of the authors and do not necessarily represent those of their affiliated organizations, or those of the publisher, the editors and the reviewers. Any product that may be evaluated in this article, or claim that may be made by its manufacturer, is not guaranteed or endorsed by the publisher.

## Footnotes

^1^ http://qiime.org/index.html

^2^ https://cran.r-project.org/bin/windows/base/old/3.4.2/

^3^ https://cran.r-project.org/web/packages/vegan/index.html

^4^ https://cran.r-project.org/web/packages/ape/index.html

^5^ https://github.com/dparks1134/STAMP

^6^ http://picrust.github.io/picrust/

^7^ https://cran.r-project.org/web/packages/ade4/index.html

^8^ https://cytoscape.org/download.html
