# Supplementary material for: Dynamic changes of the gut microbial colonization in preterm infants with different time points after birth
Source: Front Microbiol. 2023 Feb 17;14:1078426. doi: 10.3389/fmicb.2023.1078426 (PMC9983350; doi:10.3389/fmicb.2023.1078426)
Supplement: Supplementary file 1 [file Data_Sheet_1.ZIP › Supplementary Figures.docx]

**Supplementary Figure 1.** Observed taxonomic units analysis (Rarefaction Curve) of bacterial species diversity of fecal samples from all preterm infants.


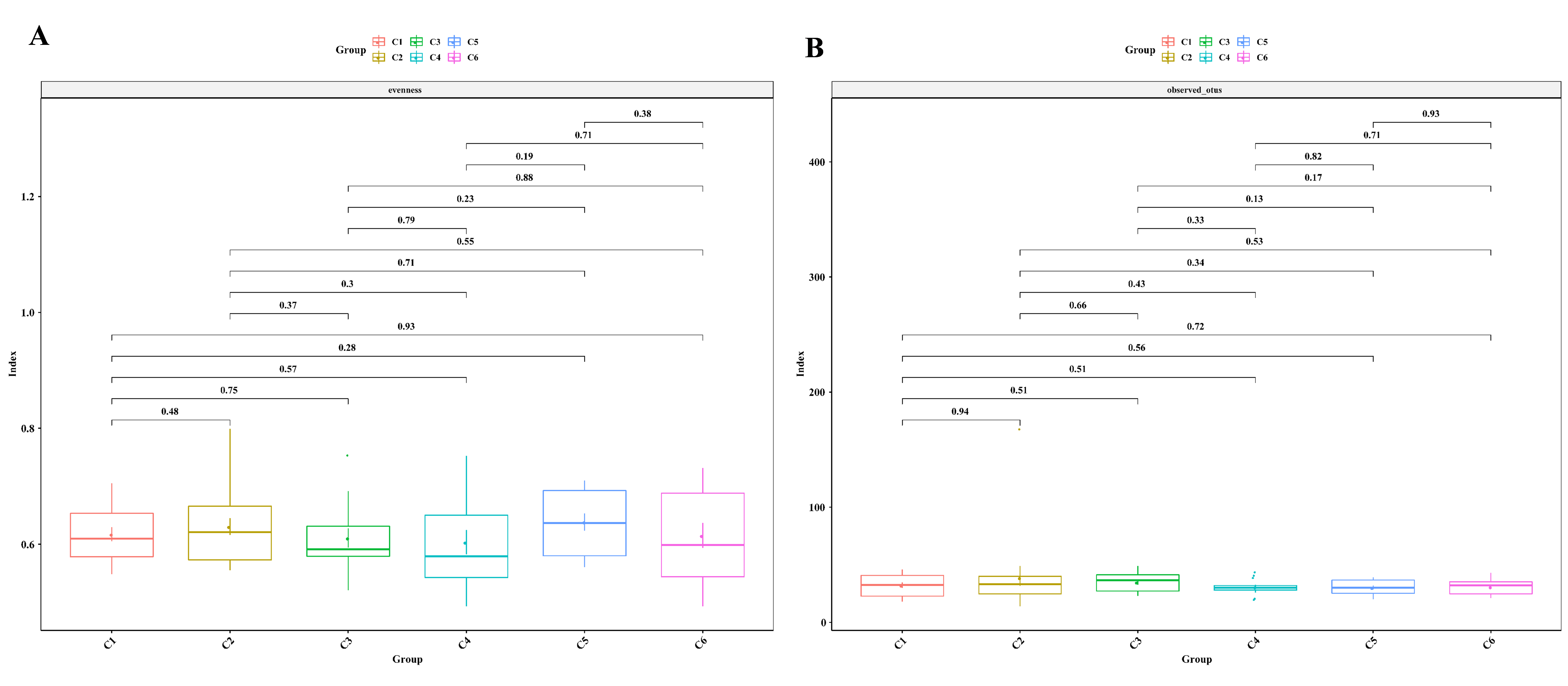


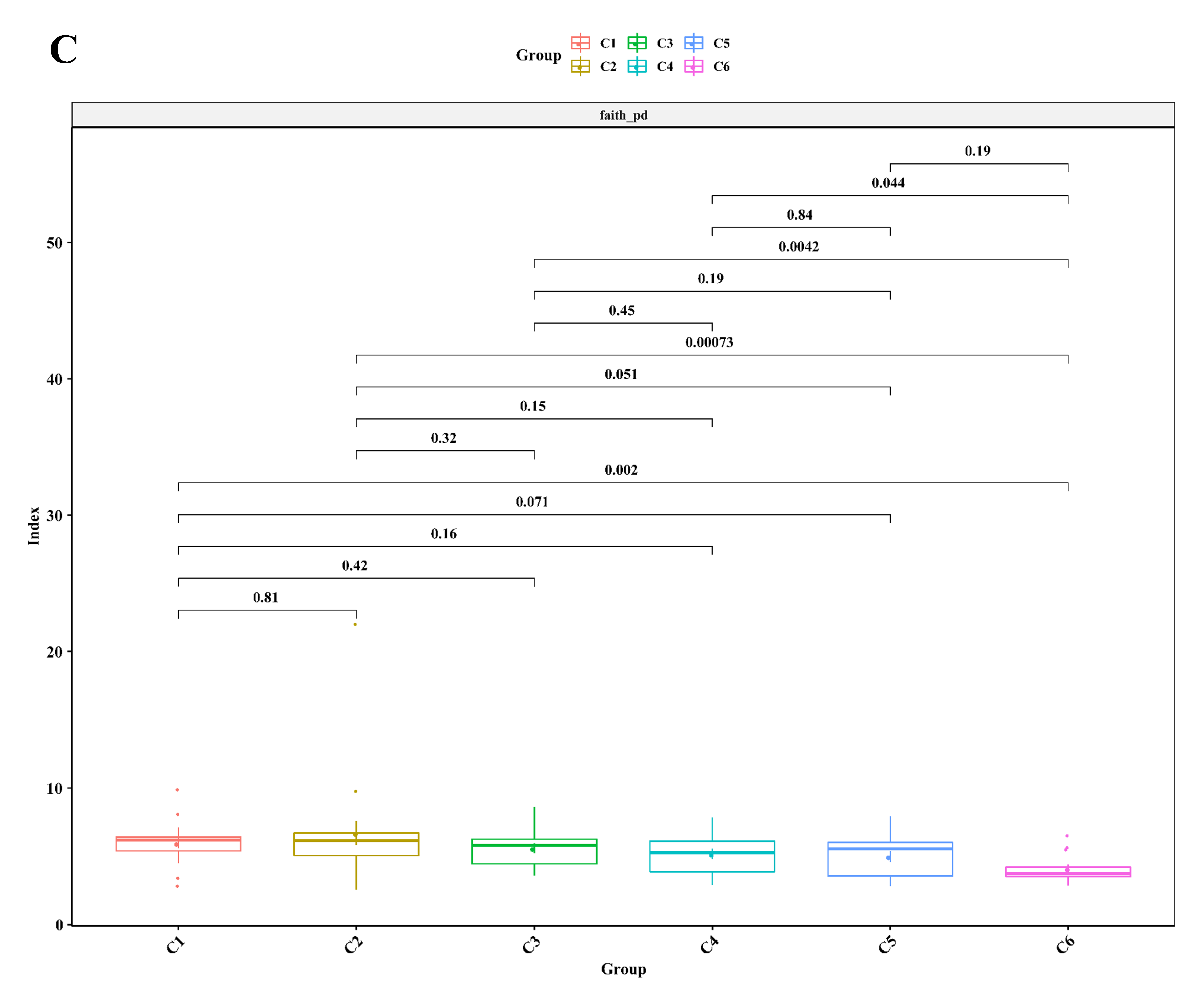


**Supplementary Figure 2**. **Alpha diversity of bacterial communities.** **(A)** Evenness **(B)** Observed OTUs **(C)** Faith_pd in the gut microbiota of preterm infants at 6 time points C1-C6 (1, 7, 14, 21, 28, and 42 days after births. One-way ANOVA was used to compare the difference across host species. Significant differences are indicated by different letters (*P < 0.05*).


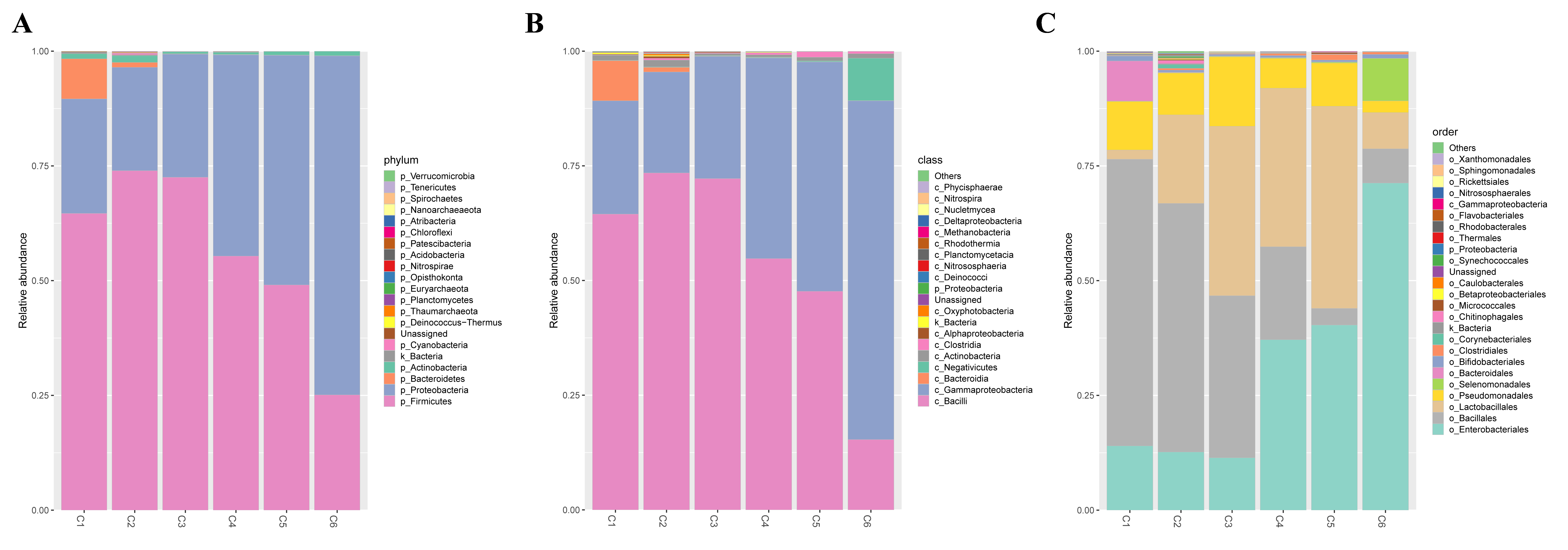





**Supplementary Figure 3.** Bar charts illustrating time period-dependent phylogenetic shifts of the top differentially abundant taxa at the phylum (A), Class (B), Order (C), Family (D), and Species levels (E). C1-C6 (1, 7, 14, 21, 28, and 42 days respectively after birth) show the groups based on different time points from birth to 42 days of age.

**

**

**

**

**Supplementary Figure 4**. Relative abundances of functions (metabolism and disease) based on KEGG classification in all groups of preterm infants i.e., C1-C6 (1, 7, 14, 21, 28, and 42 days respectively after birth) show the groups based on different time points from birth to 42 days of age.
